# Dietary *Buglossoides Arvensis* Oil Increases Circulating *n*-3 Polyunsaturated Fatty Acids in a Dose-Dependent Manner and Enhances Lipopolysaccharide-Stimulated Whole Blood Interleukin-10—A Randomized Placebo-Controlled Trial

**DOI:** 10.3390/nu9030261

**Published:** 2017-03-10

**Authors:** Natalie Lefort, Rémi LeBlanc, Marc E. Surette

**Affiliations:** 1 Department of Chemistry and Biochemistry, Université de Moncton, Moncton, NB E1A 3E9, Canada; natalie.lefort@umoncton.ca; 2 Réseau de Santé Vitalité Health Network, Centre hospitalier universitaire Dr-Georges-L.-Dumont, Moncton, NB E1C 2Z3, Canada; leblanc3430@gmail.com

**Keywords:** stearidonic acid, eicosapentaenoic acid, interleukin-10, mononuclear cells

## Abstract

*Buglossoides arvensis* (Ahiflower) oil is a dietary oil rich in stearidonic acid (20% SDA; 18:4 *n*-3). The present randomized, double blind, placebo-controlled clinical trial investigated the effects of three Ahiflower oil dosages on omega-3 polyunsaturated fatty acid (PUFA) content of plasma and mononuclear cells (MCs) and of the highest Ahiflower dosage on stimulated cytokine production in blood. Healthy subjects (*n* = 88) consumed 9.7 mL per day for 28 days of 100% high oleic sunflower oil (HOSO); 30% Ahiflower oil (Ahi) + 70% HOSO; 60% Ahi + 40% HOSO; and 100% Ahi. No clinically significant changes in blood and urine chemistries, blood lipid profiles, hepatic and renal function tests nor hematology were measured. Linear mixed models (repeated measures design) probed for differences in time, and time × treatment interactions. Amongst significant changes, plasma and MC eicosapentaenoic acid (EPA, 20:5 *n*-3) levels increased from baseline at day 28 in all Ahiflower groups (*p* < 0.05) and the increase was greater in all Ahiflower groups compared to the HOSO control (time × treatment interactions; *p* < 0.05). Similar results were obtained for α-linolenic acid (ALA, 18:3 *n*-3), eicosatetraenoic acid (ETA, 20:4 *n*-3), and docosapentaenoic acid (DPA, 22:5 *n*-3) content; but not docosahexaenoic acid (DHA, 22:6 *n*-3). Production of interleukin-10 (IL-10) was increased in the 100% Ahiflower oil group compared to 100% HOSO group (*p* < 0.05). IL-10 production was also increased in lipopolysaccharide (LPS)-stimulated M2-differentiated THP-1 macrophage-like cells in the presence of 20:4 *n*-3 or EPA (*p* < 0.05). Overall; this indicates that the consumption of Ahiflower oil is associated with an anti-inflammatory phenotype in healthy subjects.

## 1. Introduction

The enrichment of diets with *n*-3 polyunsaturated fatty acids (PUFA) achieved by the consumption of dietary oils or foods containing these fatty acids is linked to prevention of disease and positive health outcomes [1,2,3,4,5,6,7]. This benefit is particularly associated with the consumption of the 20-carbon eicosapentaenoic acid (EPA, 20:5 *n*-3) and the 22-carbon docosahexaenoic acid (DHA, 22:6 *n*-3) that are primarily found in seafood and marine oils. These long-chain *n*-3 PUFAs are preferentially incorporated into tissue phospholipids and contribute to the proper structure and function of cellular membranes. In immune cells, both *n*-6 and *n*-3 PUFA serve as substrates for lipoxygenases and cyclooxygenases, producing bioactive lipid mediators with important immunomodulatory activities [8,9]. The 20- and 22-carbon *n*-3 PUFAs in particular are precursors to lipid mediators that actively participate in the resolution of inflammation and are associated with the prevention of inflammatory diseases [10]. In addition, *n*-3 PUFAs can modulate gene expression of cytokines and adhesion molecules by interacting with the lipid-binding transcription factor peroxisome proliferator-activated receptor (PPAR) and thus also contribute to the modulation of immune and inflammatory responses [2,11,12,13].

Contrary to *n*-6 PUFA, the typical western diet does not provide the recommended amount of *n*-3 PUFA [14,15,16]. Current sources of long-chain *n*-3 PUFAs are mainly of marine origin. However, dwindling supplies of marine sources of *n*-3 PUFAs [17,18,19], and continued demands for *n*-3 PUFA sources by the aquaculture industry as a feed ingredient, coupled with the increasing desire of consumers to meet EPA and DHA recommended daily intakes have led to current efforts to identify sustainable and efficacious sources of *n*-3 PUFA. Such alternative sources include plant-derived oils that are rich in 18-carbon PUFA α-linolenic acid (ALA, 18:3 *n*-3) and stearidonic acid (SDA, 18:4 *n*-3) that are the precursors to the 20- and 22-carbon PUFA found in marine sources [20]. 

The SDA-rich Ahiflower oil (45% ALA, 20% SDA) extracted from the seed of *Buglossoides arvensis*, was recently shown in a randomized comparator-controlled trial to be more efficient than ALA-rich flaxseed oil (60% ALA) at increasing serum, erythrocyte, mononuclear cell and neutrophil EPA and docosapentaenoic acid (DPA, 22:5 *n*-3) contents [21], consistent with the poor conversion of ALA to SDA owing to a rate-limiting Δ6-desaturase, as shown by stable isotope tracer studies [22]. Additionally, Ahiflower oil contains gammalinolenic acid (GLA, 18:3 *n*-6) that also possesses anti-inflammatory properties associated with its conversion to its elongation product dihomogammalinolenic acid (DGLA, 20:3 *n*-6) [23,24]. Echium oil, SDA-soy (transgenic) and SDA-ethyl esters have also been evaluated in humans as dietary sources of SDA [25,26,27,28,29,30,31,32,33,34,35,36], however Ahiflower oil is the richest known natural source of SDA [20]. To date, the only clinical trial evaluating the efficacy of Ahiflower oil at enriching serum and circulating cells in *n*-3 PUFA was performed using 10 g of oil per day [21].

Cytokines and chemokines have been used as surrogate markers of the inflammatory response when investigating the immune-modulatory potential of interventions such as the consumption of dietary *n*-3 PUFA from marine sources [11,37,38]. Even though dietary intake of SDA leads to enhanced tissue EPA and DPA content, the impact of SDA-rich dietary oils on cytokine production has not yet been evaluated in humans. The current study describes a randomized, parallel group, double-blind, placebo-controlled clinical trial investigating the dose response to Ahiflower oil on plasma and circulating mononuclear cells *n*-3 PUFA content. Using a controlled-environment standardized method in stimulated whole blood to measure the functional immune response in humans [37], the current study is the first reported investigation of the impact of SDA-rich oil on stimulated whole blood cytokine and chemokine release in humans.

## 2. Materials and Methods

*Study approval and ethics*. Approval of the proposed use and dosages of the investigational dietary oils (Ahiflower oil and high oleic sunflower oil, HOSO) and the clinical trial design was obtained from the Natural and Non-prescription Health Products Directorate (NNHPD) at Health Canada (HC-NNHPD-213421). The ethics committees for human research of the Réseau de Santé Vitalité Health Network and the Université de Moncton approved the study procedures. This study is listed in the *clinicaltrials.gov* registry (identifier: NCT02540759).

*Study dietary oils*. Oils from high oleic sunflower (*Helianthus annuus*) seeds and from Ahiflower (*Buglossoides arvensis*) seeds were provided by Nature’s Crops International (Kensington, PE, Canada). Antioxidant (Fortium RPT 40 IP rosemary extract + ascorbyl palmitate) (Kemin Industries, Des Moines, IA, USA) and lemon flavor (FONA International, Mississauga, ON, Canada) were added, yielding 9.73 mL of refined oil per dosage unit of 10 mL for the higher dosages of HOSO (100% HOSO) and Ahiflower oil (100% Ahiflower group). Oil blends for lower intakes of Ahiflower oil contained 2.92 mL Ahiflower oil and 6.81 mL HOSO (30% Ahiflower group) and 5.84 mL Ahiflower oil and 3.89 mL HOSO (60% Ahiflower group). Fatty acid profiles of the pure and blended oils are detailed in Table 1.

*Study design*. This was a single-center (Université de Moncton), randomized, placebo-controlled, parallel-group, double-blind study in healthy men and women. With an expected minimally significant change of 40% in plasma EPA concentrations [29], a standard deviation of 25%, the level of significance set at 0.05 and a desired power of 0.95, the calculated sample size was nine subjects per group. In order to account for dropouts and non-compliance, 24 subjects were enrolled per group in the 100% HOSO and 100% Ahiflower groups and 20 subjects in the 30% and 60% Ahiflower groups. More subjects were recruited in the 100% oil groups to maximize discernment of changes in cytokines and chemokines following whole blood lipopolysaccharide (LPS) stimulation. LPS stimulation was completed only on the 100% oil groups. Recruitment began in September 2015 and ended in February 2016. The study was completed in March 2016 when 20 or 24 participants in each intervention had completed the study. 

Inclusion criteria included being between 18 and 65 years of age and having a body mass index (BMI) of 18–39.9 kg/m^2^. Exclusion criteria were: pregnancy or lactation, medical conditions such as an active peptic ulcer, inflammatory bowel disease or gastrointestinal bleeding that could influence absorption, metabolism or excretion of the study supplement, a history or presence of significant renal, hepatic, gastrointestinal, pulmonary, biliary, neurological or endocrine disorders, the consumption of fish oil or other *n*-3 or *n*-6 PUFA supplements/drugs within one month of beginning the trial, consumption of fatty fish (salmon, herring, mackerel, albacore tuna, and sardines) more than twice a month in the month preceding beginning the trial and unwillingness to avoid PUFA supplements and seafood throughout the study period, and chronic administration of anti-inflammatory medications (including asthma, allergy and pain medications). All inclusion and exclusion criteria can be found in Supplementary Table S1.

**Figure 1:**
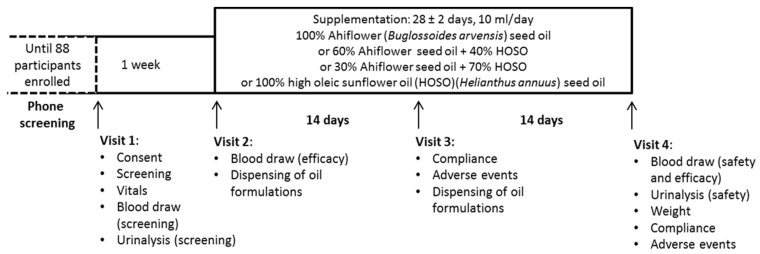
Clinical trial timeline and activities completed at each visit.

Eligible subjects met the clinical research team at the Université de Moncton. During the first visit, they completed a consent form, provided a fasting blood sample and a urine sample, and vital sign measurements were documented (Figure 1).

Following Visit 1, subjects were excluded from enrolment in the event of clinically significant abnormal laboratory test results including, but not limited to, low density lipoprotein (LDL)-cholesterol ≥4.1 mM, triglyceride ≥3.95 mM, fasting creatinine ≥1.5 mg/dL, alanine aminotransferase or aspartate aminotransferase ≥1.5X the upper limit of normal, uncontrolled hypertension (resting systolic blood pressure (BP) ≥160 mmHg or diastolic BP ≥100 mmHg) and hemoglobin A1c (HbA1c) ≥6.0. Subjects were enrolled into the study following review of the screening documents and approval by the study physician.

Approximately 7–10 days following Visit 1, enrolled subjects were randomly allocated to one of four supplementation groups (100% HOSO (HOSO group), 100% Ahiflower (100% group), 60% Ahiflower + 40% HOSO (60% group) and 30% Ahiflower + 70% HOSO (30% group)) at Visit 2 by the study coordinator. A block randomization method using an online algorithm (random.org) with two blocks of 20 and two blocks of 24 subjects generated a participant enrollment order. The study coordinator and the study nurse were aware of which groups were assigned 100% oils and which groups were assigned oil blends, but not the composition of each individual formulation. This was necessary to identify which participant underwent the whole blood inflammation response analysis. The study physician was blinded until all safety and efficacy data were compiled and pre-determined statistical analyses completed. The identity of the oil formulations was kept at the bottling facility. The four groups consumed 10 ml/day of one of four oil formulations (100% HOSO, 100% Ahiflower, 60% Ahiflower or 30% Ahiflower). Subjects were provided one bottle of oil at Visit 2 for days 1–14 and a second bottle at Visit 3 for days 15–28. Subjects were instructed to store the bottles of oil in the refrigerator and to measure (using a single-use oral syringe) and consume 10 mL of oil daily, preferably at the same time of day, with a meal, for 28 ± 2 days. They were given the option to consume the oil directly from the syringe or to add to a food compatible with oils such as yogurt, pasta sauce or a piece of bread. Visit 2 included a blood draw (following a 10 to 12 h fast) for baseline fatty acid analyses. Participants assigned pure oils provided an additional 2 mL of blood for inflammatory response analyses at Visits 2 and 4. At Visit 3, reported adverse events (AE) were documented. Visit 4 included a blood draw (following a 10 to 12 h fast) and a urine test for measurement of safety and efficacy endpoints as well as recording of AE. Safety endpoint materials (blood and urine samples) were promptly forwarded to the Centre Hospitalier Universitaire Dr-Georges-L.-Dumont clinical laboratory in Moncton, NB, for processing and analyses.

*Compliance*. Adherence to the dosage regimen was assessed by documenting residual oil volumes in the bottles returned by the participant. If less than 80% of the doses over the four-week period or if less than 80% of the doses over days 15–28 were consumed, the participant was deemed non-compliant and their data were not included in the efficacy (fatty acid and inflammatory response) analyses.

*Recording of adverse events and reactions*. Subjects were informed of their responsibility to report all physical and psychological changes during the study and up to 28 days after the last dose. AE recall was facilitated by using open-ended questioning such as “How have you felt since your last visit?” and “Have you had any new or changed health problems since you were last here?” Clinical laboratory values more than 1.5X the upper normal range were also considered AEs. AEs were graded in intensity (mild, moderate or severe), in severity (graded using the Common Terminology Criteria for Adverse Events version 4.03) [39] and causality (unrelated, unlikely, possible, probable, definitely related) promptly by the study physician. AEs possibly, probably or definitely related to the consumption of a dietary oil were defined as adverse reactions (AR). All follow-up to reported AEs and ARs were promptly provided by the study physician.

*Blood fractionation for efficacy endpoint measurements*. The efficacy endpoints were plasma and mononuclear cell eicosatetraenoic acid (ETA, 20:4 *n*-3), EPA and DPA (% of total fatty acids), plasma ETA, EPA and DPA concentrations (μmol/L), as well as LPS-stimulated whole blood cytokine and chemokine (interleukin-6 (IL-6), monocyte chemoattractant protein-1 (MCP-1), tumor necrosis factor-alpha (TNF-α), interferon-gamma (IFN-γ), interferon-alpha (IFN-α), interleukin-1beta (IL-1β), interleukin-33 (IL-33), interleukin-23 (IL-23), interleukin-18 (IL-18), interleukin-10 (IL-10), interleukin-12 p70 (IL-12p70), interleukin-17A (IL-17A), interleukin-8 (IL-8) and IL-1 receptor antagonist (IL-1Ra)) concentrations (in pg/mL).

Briefly, 20 mL of whole blood were collected on heparin. A 2 mL aliquot was centrifuged (1020× *g*, 15 min) and resulting plasma was collected and centrifuged (3000× *g*, 20 min) to remove platelets resulting in platelet-free plasma. Cells from remaining whole blood (18 mL) were collected following dextran sedimentation and centrifugation on a lymphocyte separation medium cushion [40]. Briefly, three volumes of whole blood were diluted with one volume of Hank’s buffered salt solution (HBSS) and one volume of 3% Dextran in HBSS to encourage erythrocytes to settle (60 min). The resulting cell suspension (top-layer) was diluted with one volume of HBSS and cells were recovered by centrifugation (200× *g*, 10 min). Cells resuspended in HBSS were placed on a cushion of Lymphocyte Separation Medium (density: 1.077 g/mL) (Wisent, St-Bruno, QC, Canada) and centrifuged (800× *g*, 20 min) at room temperature. The buffy coat containing mononuclear cells was collected from the interface, washed twice and resuspended in HBSS. Plasma (diluted 1:4 in HBSS) and mononuclear cells were immediately added to 3.75 volumes of a solution of CHCl_3_:MeOH (1:2) and stored at −20 °C for future lipid extraction.

*Lipid extraction and fatty acid analysis*. The internal standard di-heptadecanoyl-PC (Matreya LLC, State College, PA, USA) was added to samples stored in CHCl_3_:MeOH, then lipids were extracted using the Bligh and Dyer method [41]. Fatty acid methyl esters (FAMEs) were prepared and analyzed by gas chromatography as previously described [21]. Briefly, the extracts were saponified with 0.5 M KOH in methanol (100 °C, 15 min) and FAMEs were prepared by adding 14% BF_3_ in methanol and heating at 100 °C for 10 min. FAMEs were extracted in hexane and quantified by gas chromatography with flame ionization detection (GC-FID) using a 30 m BPX-70 column (0.25-mm internal diameter, 0.25-μM film thickness) (SGE Analytical Science, Pflugerville, TX, USA) on a Thermo Trace gas chromatograph (Thermo Electron Corporation, Mississauga, ON, Canada). FAME standards (Nu Chek Prep, Elysian, MN, USA) were used for the determination of FAME peak retention times and for generation of individual FAME standard curves. The intra-assay precision (% relative SD) of this method for samples containing 50 μg of individual fatty acids per 100 μL plasma is approximately 2% [24].

*Whole blood stimulation*. Immediately after puncture for blood draw, a TruCulture (Myriad RBM, Austin, TX, USA) tube containing LPS (10 ng/mL) was filled with 1 mL of blood. A second TruCulture tube devoid of stimulant (control) was filled with 1 mL of blood. TruCulture tubes were inverted 3 times, and incubated at 37 °C with 5% CO_2_ for 23.5–27 h. The duration of the incubation for the baseline measurement was applied to the post-supplementation sample for each subject. Supernatants were collected and stored in aliquots at −80 °C.

*Detection of cytokines and chemokines*. IL-1β, IFN-α, IFN-γ, TNF-α, MCP-1 (chemokine (C-C) motif ligand 2), IL-6, IL-8 (C-X-C motif chemokine ligand 8), IL-10, IL-12p70, IL-17A, IL-18, IL-23, IL-33 and IL-1Ra were quantified in the Truculture tube supernatants using the Human Inflammation Panel and a custom-made IL-1Ra (LEGENDplex™, BioLegend, San Diego, CA, USA) bead-based immunoassays. This panel was chosen since it contained several cytokines and chemokines sensitive to LPS stimulation with low interindividual variance, high reproducibility and reliability using the TruCulture method [37]. Briefly, supernatant was added to pre-mixed beads, detection antibodies and proprietary assay buffer and incubated for 2 h at 600 rpm at room temperature. Streptavidin–phycoerythrin was added to each sample and the assay plate was incubated for 30 min at 600 rpm. A standard curve was generated (from 2.4 pg/mL to 10,000 pg/mL for all cytokines, except for IL-33 and IL-1Ra; the standard curve for IL-33 ranged from 12.2 to 50,000 pg/mL and for IL-1Ra from 122 to 500,000 pg/mL) using the provided Human Inflammation Panel Standard cocktail. Streptavidin–phycoerythrin conjugate intensity was detected using an FC500 flow cytometer (Beckman Coulter). A reversed cubic curve fit with logarithmic axes was used to generate standard curves for each cytokine and chemokine. 

*Differentiation and stimulation of the THP*-*1 monocytic cell line*. THP-1 monocytic cells (1 × 10^6^ cells/mL in RPMI media containing 10% heat-inactived FBS, I-FBS) were differentiated into macrophages with 10 µM phorbol 12-myristate 13-acetate (PMA) for 48 h, yielding THP-1 M0 cells. Cells were washed three times with RPMI media. Cells were then polarized to M2 macrophages by exposure to 20 µg/mL interleukin-4 for 48 h. Simultaneously, one of the following treatments was applied: 0.5% ethanol, 50 µM SDA, 50 µM ETA or 50 µM EPA. Free fatty acids dissolved in ethanol were added directly to culture medium. Subsequently, M2 macrophages were stimulated with 10 µg/mL LPS or its diluent (1% phosphate-buffered saline (PBS)) for 24 h. Ethanol and fatty acid treatments were continued during LPS stimulation. Supernatant was recovered and stored at −80 °C until IL-10 analysis using a bead-based immunoassay (LEGENDplex™, BioLegend, San Diego, CA, USA) as described above.

*Statistical analyses*. Linear mixed models (LMM) were fitted to determine whether the percentage and/or the concentration of the following polyunsaturated fatty acids differed between the 100% HOSO, 100% Ahiflower, 60% Ahiflower and 30% Ahiflower groups at baseline and at day 28 after commencement of supplementation in plasma and mononuclear cells: ALA, SDA, ETA, EPA, DPA, DHA and dihomo-gamma-linolenic acid (DGLA, 20:3 *n*-6). The percentage of ALA, SDA, ETA, EPA, DPA, DHA and DGLA in plasma or mononuclear cells or the concentrations of ALA, SDA, ETA, EPA, DPA and DHA in plasma were used as the response in each LMM. The variables group (100% HOSO, 100% Ahiflower, 60% Ahiflower and 30% Ahiflower), time, time × treatment interaction, as well as the covariates age, gender and weight were defined as predictors, and participant identification number was defined as a random variable. LPS-stimulated cytokine and chemokine concentrations were analyzed in an identical manner; however, the variables group only included 100% HOSO and 100% Ahiflower. Plasma and mononuclear cell percentages of ALA, EPA, DPA, DHA and DGLA and concentrations of ALA, EPA, DPA and DHA in plasma, and cytokine and chemokine concentrations were log-transformed to respect assumptions of homoscedasticity and normality of residuals. Because the assumption of homoscedasticity of residuals was not met in the analyses of the percentages and the concentrations of SDA and ETA, a one-way analysis of variance followed by a Tukey’s multiple comparison test was performed on the change in the percentages in plasma and mononuclear cells and the plasma concentrations of SDA and ETA between days 0 and 28. 

Unless otherwise mentioned, all analyses respected the assumptions of normality and homoscedasticity of residuals and were performed with R 3.2.5 [42], except the SDA and ETA analysis, which was completed using GraphPad Prism 7.00 (GraphPad Software, La Jolla, CA, USA). 

## 3. Results

### 3.1. Subject Characteristics

Responders (*n* = 164) to the study advertisement were screened using a questionnaire. Eligible candidates (*n* = 113) were invited for Visit 1. Of these, 88 (*n* = 24 for 100% HOSO and 100% Ahiflower groups; *n* = 20 for 30% and 60% Ahiflower groups) men and women were enrolled into the study. Baseline anthropometric and clinical characteristics are listed in Table 2. All intervention groups had similar mean age, mean weight, mean BMI and gender distribution.

#### 3.1.1. Retention and Adherence to Study Protocol

**Figure 2:**
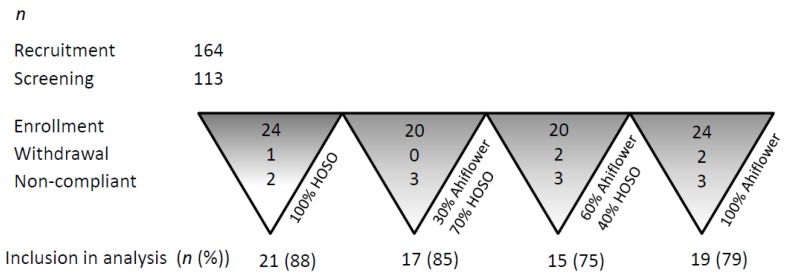
Participant recruitment, retention and adherence to protocol dosage and activities.

The number of participants included in the efficacy analyses in each group is listed in Figure 2.

The single withdrawal from 100% HOSO group was due to a serious AE (unlikely related to consumption of a dietary oil). One participant from 100% Ahiflower group withdrew consent and a second participant withdrew consent due to an AE (acid reflux and nausea). All participants in the 30% Ahiflower group completed the study. One participant from 60% Ahiflower group withdrew consent prior to starting supplementation and a second participant was lost to follow-up. Two participants from the 100% HOSO group (12%) and three from each of the other groups (15%–25% of participants) were excluded from efficacy analyses due to lack of compliance to the dosage protocol.

#### 3.1.2. Safety Outcomes

No clinically significant changes in fasting blood chemistry, hematology, fasting lipid profiles and hepatic and renal function tests following the supplementations were observed in participants in the four intervention groups. Ten AEs were reported by 7 participants in the 100% HOSO group, 11 AEs were reported by 8 participants in the 30% Ahiflower group, 20 AEs were reported by 9 participants in the 60% Ahiflower groups and 17 AEs were reported by 7 participants in the 100% Ahiflower group. One AR was reported in the 100% HOSO group, one AR was reported in the 30% Ahiflower group, 6 ARs were reported by 3 participants in the 60% Ahiflower groups and 7 ARs were reported by 5 participants in the 100% Ahiflower group. All AE and AR were classified as mild in intensity and graded low severity.

### 3.2. Fatty Acid Analyses

#### 3.2.1. α-Linolenic Acid (18:3 *n*-3)

In plasma, ALA as a percentage of total fatty acids increased significantly compared to baseline in the 30%, 60% and 100% Ahiflower groups and did not change in the 100% HOSO group (Table 3).

The increase was dose-dependent with the 30% Ahiflower group showing the smallest increase and the 100% Ahiflower group displaying the highest increase. The changes in % ALA content were significantly different between all groups (time × treatment effect). In mononuclear cells, a significant increase in ALA content compared to baseline was observed in the 60% and 100% Ahiflower groups (Table 4). The change in ALA content was affected by treatment only in the 100% Ahiflower group (time × treatment effect).

No difference in plasma ALA concentration (μmol/L) compared to baseline was observed following 28 days of supplementation in the 100% HOSO and 30% Ahiflower groups, whereas ALA concentration increased in the 60% and 100% Ahiflower groups. The amplitude of the increase was not different between the 60% and 100% Ahiflower groups (Supplementary Table S2).

#### 3.2.2. Stearidonic Acid (18:4 *n*-3) and Eicosatetraenoic Acid (20:4 *n*-3)

SDA was detected in 1 (100% HOSO), 14 (30% Ahiflower), 16 (60% Ahiflower) and 21 (100% Ahiflower) participants following the 28-day supplementation. In plasma, the change in SDA content as a percentage of total fatty acids was significantly greater in all Ahiflower groups compared to the 100% HOSO control, with the 60% and 100% Ahiflower groups displaying a greater increase compared to the 30% Ahiflower group (Table 3). In mononuclear cells, SDA content was modified by supplementation uniquely in the 100% Ahiflower group (Table 4). SDA plasma concentration changes (μmol/L) were significantly greater in the 60% and 100% Ahiflower groups. Both these groups displayed a greater increase than the 30% Ahiflower group, which displayed a greater increase than the 100% HOSO control group (Supplementary Table S2).

The ETA changes in percentage of total fatty acids and concentration (μmol/L) in the 60% and 100% Ahiflower groups was significantly greater than in the 30% Ahiflower and 100% HOSO groups in plasma (Table 3 and Supplementary Table S2). A treatment effect was observed in mononuclear cells in the 60% and 100% Ahiflower oil groups, with the 100% Ahiflower group displaying a higher increase than the 60% Ahiflower group (Table 4).

#### 3.2.3. Eicosapentaenoic Acid (20:5 *n*-3)

EPA content as a percentage of total fatty acidsincreased significantly compared to baseline in the 30% Ahiflower, 60% Ahiflower and 100% Ahiflower groups following 28-day supplementation in both plasma and mononuclear cells (Table 3 and Table 4). EPA content decreased compared to baseline in the 100% HOSO control group in mononuclear cells. EPA increased significantly in plasma and in mononuclear cells in all Ahiflower groups compared to the 100% HOSO group (time × treatment interaction). The extent of the increase was greatest in the 60% Ahiflower and 100% Ahiflower group, with no difference between these two groups in both plasma and mononuclear cells. The 100% Ahiflower group displayed a greater increase in EPA than the 30% Ahiflower group in both plasma and mononuclear cells.

**Figure 3:**
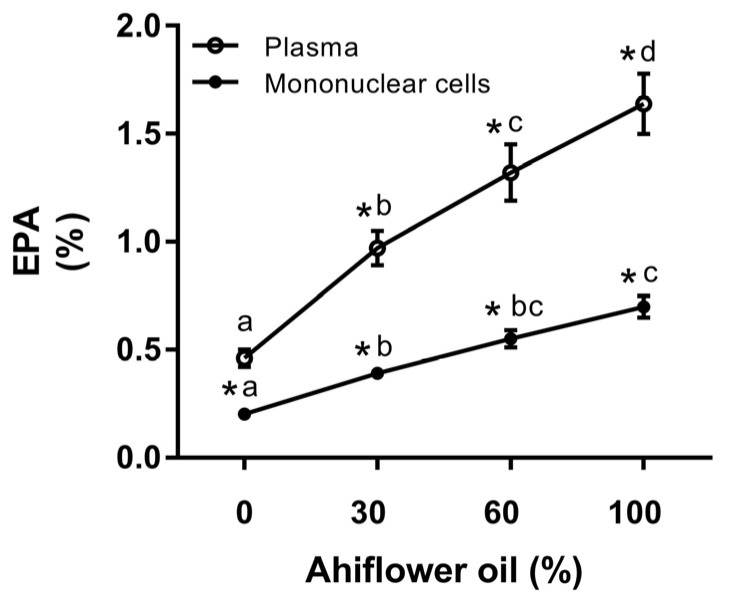
The change in the eicosapentaenoic acid (EPA) content of plasma and mononuclear cells with Ahiflower oil intake. Values are expressed as the percentage of total fatty acids at day 28 for each dietary group. Values are the mean ± SEM. Linear mixed models (LMM) were fitted to identify significant differences between treatments. Groups with different letters are significantly different (*p* ≤ 0.05). * *p* ≤ 0.05 vs. baseline values.

In order to illustrate the dose-response changes in EPA that were impacted by Ahiflower oil consumption, EPA content as a percentage of total fatty acids in plasma and in mononuclear cells for the different dietary groups is shown in Figure 3. As can be seen, the response to Ahiflower intake on EPA content is linear, suggesting that at these intake dosages the conversion of SDA to EPA coupled with its incorporation into plasma and mononuclear cells has not attained saturation.

In plasma, EPA concentration (μmol/L) increased compared to baseline in all Ahiflower groups. EPA also increased significantly in all Ahiflower groups compared to the 100% HOSO control group (time × treatment interaction), with the 60% and 100% Ahiflower groups displaying the greatest increase (Supplementary Table S2).

#### 3.2.4. Docosapentaenoic Acid (22:5 *n*-3) and Docosahexaenoic Acid (22:6 *n*-3)

Plasma DPA accrual as percentage of total fatty acidsincreased significantly compared to baseline in the 60% and 100% Ahiflower groups (Table 3). The DPA increase in the 60% and the 100% Ahiflower oil groups was also significant compared to the 100% HOSO control group (time × treatment interaction), with the 100% Ahiflower group displaying the greatest change. In mononuclear cells, DPA increased significantly compared to baseline in all Ahiflower groups, and decreased in the 100% HOSO group. All Ahiflower supplementations led to a greater change in DPA compared to 100% HOSO, with the 60% and 100% Ahiflower groups displaying the greatest change (Table 4). DPA concentrations (μmol/L) in plasma increased in the 100% Ahiflower group compared to baseline and compared to all other groups, with no change observed in the other groups (Supplementary Table S2). 

Plasma DHA content, both percentage of total fatty acidsand concentration, was unchanged from baseline in all supplementation groups (Table 3 and Supplementary Table S2). In mononuclear cells, DHA content decreased significantly from baseline in 100% HOSO, 60% Ahiflower and 100% Ahiflower groups. The decreases were not different between these three groups (Table 4).

#### 3.2.5. Dihomo-γ-Linolenic Acid (20:3 *n*-6)

Since Ahiflower oil contains GLA, the content of its elongation metabolite DGLA was measured since it has been shown to possess anti-inflammatory properties [23,24]. DGLA content in plasma remained stable in all supplementation groups. However, in mononuclear cells, DGLA content increased significantly from baseline in the 100% Ahiflower group, and this increase was different from the 100% HOSO and the 30% Ahiflower groups (time × treatment interaction).

### 3.3. Cytokine Analyses

#### 3.3.1. Whole Blood Cytokine and Chemokine Response to LPS

**Figure 4:**
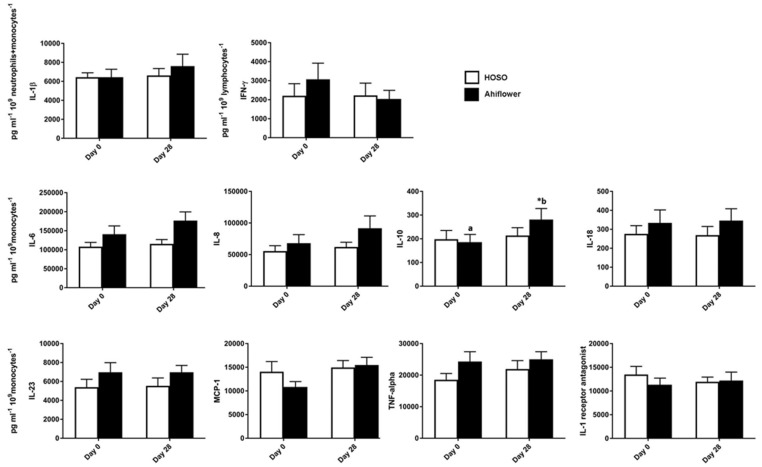
LPS-stimulated cytokine and chemokine production in whole blood. Whole blood was incubated with LPS (10 ng/mL) for 24 h prior to supplementation and after 28 days of dietary supplementation with the indicated oils. Cytokine and chemokine concentrations were measured in the supernatant using the Human Inflammation Panel (LEGENDplexTM, BioLegend, San Diego, CA, USA) and were normalized to their main cell producer. HOSO group *n* = 21, Ahiflower group *n* = 19. Data are mean ± SEM. * Different from baseline, *p* < 0.05; conditions with different letters were different at Day 28 as determined by LMM, *p* < 0.05. IL-1β = interleukin-1beta; IFN-γ = interferon-gamma; IL-6 = interleukin-6; IL-8 = interleukin-8; IL-10 = interleukin-10; IL-18 = interleukin-18; MCP-1 = monocyte chemoattractant protein-1; IL-23 = interleukin-23; TNF-α = tumor necrosis factor-alpha; IL-1 = interleukin-1.

In the absence of LPS, cytokine and chemokine concentrations were near or below limits of detection for all participants in the 100% HOSO (*n* = 21) and 100% Ahiflower (*n* = 19) groups. Of the 14 cytokines and chemokines tested, 10 responded to the LPS challenge (Figure 4), data are not presented for the non-responding cytokines: IFN-α, IL-12p70, IL-17A, and IL-33. Cytokine and chemokine concentrations were normalized to the relevant cell counts. IL-6, MCP-1, TNF-α, IFN-γ, IL-1β, IL-23, IL-18, IL-8 and IL-1Ra normalized concentrations were not different between 100% HOSO and 100% Ahiflower groups. Normalized concentrations of IL-10 increased significantly in the 100% Ahiflower group (*p* = 0.0006), and the normalized concentrations between groups were significantly different at day 28 (*p* = 0.04) (Figure 4).

#### 3.3.2. PUFA Modulation of IL-10 Production by M2-Like THP-1 Macrophages

**Figure 5:**
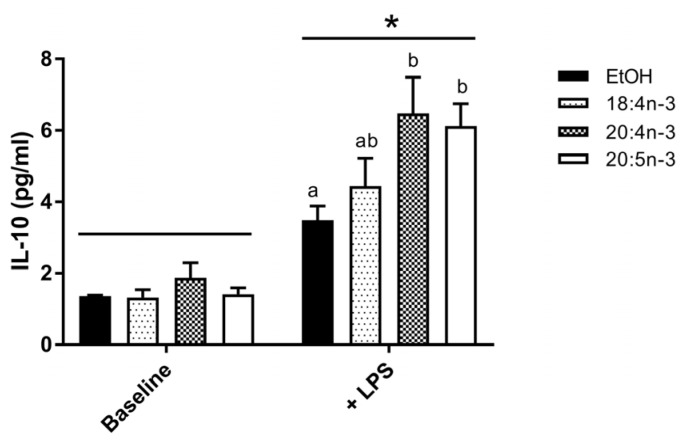
IL-10 production in differentiated THP-1 M2 macrophages. THP-1 M0 macrophages were exposed to SDA, ETA and EPA (50 µM) during differentiation to M2-like macrophages. PUFA-exposed M2-like macrophages were then stimulated with 1% phosphate buffered saline (Baseline) or lipopolysaccharide (10 µg/mL, +LPS) for 24 h. Cell media was collected and IL-10 concentration determined by bead-based immunoassay. Data are mean ± SEM, *n* = 3–5 per condition. Two-way ANOVA followed by Tukey’s multiple comparison test probed for PUFA and LPS stimulation effects. * LPS stimulation effect, *p* < 0.01; PUFA conditions without a common letter are significantly different, *p* < 0.05.

To investigate the impact of fatty acids on IL-10 production in M2 macrophages, human THP-1 M0 cells were differentiated into M2-like cells in the presence or absence of SDA, ETA and EPA and stimulated with LPS. Exogenous fatty acids were effectively incorporated into cells in all groups with a 6- to 7-fold increase in cellular *n*-3 PUFA content compared to controls. Incubation with LPS increased IL-10 production by M2-like macrophages regardless of PUFA treatment (*p* < 0.01) (Figure 5). Moreover, LPS-induced IL-10 production was significantly increased in cells incubated with ETA and EPA, but not 18:4 *n*-3, compared to EtOH control.

## 4. Discussion

Plant-derived oils have garnered recent interest as sustainable alternatives to marine-derived sources of dietary omega-3 PUFA. Although several plant seed oils such as flaxseed (~60% ALA) and *Camelina sativa* (~40% ALA) oils are rich in ALA, dietary plant oils containing SDA were suggested to be a more effective source of *n*-3 PUFA because of the better conversion of SDA to EPA and DPA compared to that of dietary ALA. A recent controlled clinical trial directly comparing SDA-rich Ahiflower oil to ALA-rich flaxseed oil confirmed this superior conversion in three different blood cell types and in plasma [21], while a recent report comparing different clinical trials investigating echium and flaxseed oils came to the same conclusion [31]. 

The current study investigated the dose response of Ahiflower oil intake on plasma and mononuclear cell fatty acid content and on stimulated cytokine and chemokine production in whole blood. The only previous placebo-controlled dose response trial investigating SDA consumption was conducted using SDA-ethyl esters and only measured erythrocyte fatty acids [29]. In the present study, the consumption of Ahiflower oil resulted in a significant dose-related increase in EPA content compared to baseline content and compared to control in both plasma and mononuclear cells after 4 weeks of dietary supplementation. This increase was significant at all doses in both plasma and mononuclear cells with an increase measured even at the lowest daily dose of 3 g Ahiflower oil, which delivered approximately 0.44 g of SDA per day. Similarly, mononuclear cell DPA content was also significantly increased at the lowest dose whereas plasma DPA was significantly impacted at the higher daily doses. This indicates that immune cells are sensitive to low doses of SDA with an accumulation of its elongation/desaturation products. Accordingly, the only other trial investigating such a low dose of SDA reported an intake of 0.43 g per day of SDA-ethyl esters with no significant impact on erythrocyte PUFA content [29], while in a trial in which mononuclear cells were evaluated, an intake of 1 g SDA per day as echium oil more than doubled the EPA content, similar to the 60% Ahiflower group in the current study [34].

It should not be overlooked that Ahiflower oil also delivers a significant amount of dietary ALA that can also contribute to tissue *n*-3 PUFA content. However, the contribution of dietary ALA to tissue content of longer chain *n*-3 PUFA is likely negligible considering that Ahiflower oil, which contains a near equivalent content of ALA as flaxseed oil, is significantly more effective at impacting tissue *n*-3 PUFA content [21]. Consistent with previous studies, plasma and mononuclear cell SDA content was not impacted significantly, indicating that SDA is preferably metabolized rather than being acylated into membrane phospholipids. Also, as in most previous trials investigating oils that contain SDA [20], DHA content did not increase, suggesting that the delta-6 desaturase-catalyzed transformation necessary for DHA production is not active in populations consuming typical western diets. 

The consumption of preformed long chain *n*-3 PUFA from marine oils has been associated with changes in inflammatory mediator production, partly explaining their beneficial impact on health parameters. Indeed, in addition to being precursors to pro-resolving lipid mediators [10], consumption of long chain *n*-3 PUFA have been shown to decrease ex vivo production of inflammatory cytokines in some but not all human studies [11,38,43,44]. Although SDA-containing oils are not as potent as marine oils in modifying tissue PUFA content [28,29,33,45], the changes in *n*-3 PUFA associated with the consumption of Ahiflower oil are nevertheless qualitatively similar to those measured following the consumption of marine oils with increases in EPA and DPA content. Surprisingly, there are no reports of measurements of inflammatory cytokines in subjects following the consumption of SDA-containing oils. In order to evaluate the potential impact of Ahiflower oil intake on inflammatory responses, a recently-reported method was used in which a functional immune response is induced in whole blood under optimized conditions, providing a reliable and reproducible assay system that permits standardized immunophenotyping [37].

As expected, several cytokines were produced in blood in response to LPS stimulation [37]; whereas others in the panel of measured cytokines remained below the limit of detection. This latter result is not altogether surprising since IFN-alpha is not produced in response to LPS [46], while IL-33 is produced by monocytes in response to LPS but is not released by the cells [47]. Amongst the 10 cytokines that were induced in stimulated whole blood, IL-10 was the only cytokine that was significantly impacted by Ahiflower oil consumption with a 45% increase compared to baseline. Fatty acids modulated by Ahiflower oil likely do not impact transcription factors involved in the transcription of the nine cytokines for which the abundance was unchanged following Ahiflower oil supplementation. IL-10 is an anti-inflammatory cytokine produced mainly by immunosuppressive M2-like monocyte/macrophages [48]. M2 cells, amongst other roles, dampen inflammation and promote tissue remodeling and angiogenesis [49]. IL-10 in turn can polarize M2 cells towards an M2c phenotype which is implicated in the deactivation of inflammation [49]. For example, IL-10 lowers the expression of major histocompatibility complex class II [50] and co-stimulatory molecules CD86 [51] on antigen-presenting cells impeding the presentation of antigens to T cells. It is noteworthy that the abundance of IL-10 can predict the severity of several human diseases with an inflammatory etiology, with low circulating IL-10 suggesting a greater disease severity (reviewed in [48]). It is difficult to extrapolate the present increase in IL-10 observed following consumption of Ahiflower oil to a clinical impact on disease. However, juvenile rheumatoid arthritis patients homozygous for the GCC haplotype in the promoter region of the IL-10 gene have less disease severity and show 50% higher IL-10 production in blood following LPS stimulation compared to the ATA haplotype [52]. This suggests that moderate changes in IL-10 production as shown in the present study can be associated with disease severity. Therefore, a dietary product which can increase circulating IL-10 could hold promise for the modulation of chronic inflammation. 

The impact of dietary Ahiflower oil on IL-10 concentrations is consistent with some previous studies investigating long-chain *n*-3 PUFAs. Parenteral administration of fish oil in patients with severe acute pancreatitis increases IL-10 [53] and the consumption of fish oil (1.8 g/day, 3 months) by obese patients with dyslipidemia led to higher circulating IL-10 concentrations [54]. Subsequently, EPA was identified as a positive regulator of IL-10 secretion in cultured THP-1 M0 monocytes, and transcriptional upregulation of IL-10 by this PUFA was dependent on peroxisome proliferator-activated receptor gamma (PPARγ) [54], the main PPAR expressed in immune cells and which is responsive to PUFA [55]. In accordance, incubation with EPA and DHA increased IL-10 positive human monocytes following LPS stimulation [56]. The current study supports a role for EPA in stimulating IL-10 production in THP-1 M2-like macrophages. In addition, we propose that the SDA elongation product ETA is also capable of increasing IL-10 production in these cells. This is the first report of the positive effect of the consumption of SDA-rich dietary oil on IL-10, an anti-inflammatory cytokine displaying a deficiency in human autoimmune diseases [48].

Overall, this placebo-controlled trial showed a dose-dependent enrichment of plasma and circulating mononuclear cells with 20-carbon (ETA and EPA) and 22-carbon (DPA) *n*-3 PUFAs following consumption of SDA-rich Ahiflower oil, and that a dose as low as 3 g per day resulted in significant plasma and mononuclear cell enrichment with EPA after 4 weeks of dietary supplementation. The use of a standardized method to measure a functional immune response in whole blood revealed that Ahiflower oil consumption was also associated with the increased production of the anti-inflammatory cytokine IL-10. This is the first investigation of an immune response following the consumption of SDA-containing oil, and indicates that dietary oils such as Ahiflower oil may share immune modulating properties that are typically associated with the consumption of marine oils. Future studies should be conducted to determine the potential impact of such dietary oils on the biosynthesis of pro-resolving mediators of inflammation and on objective measures of chronic disease.

## Figures and Tables

**Table 1:** Fatty acid composition of Ahiflower and high oleic sunflower oil (HOSO) formulations used for supplementation. GLA = gammalinolenic acid; ALA = α-linolenic acid; SDA = stearidonic acid; PUFA = polyunsaturated fatty acids; ND = not detected.

	0% Ahiflower 100% HOSO	30% Ahiflower 70% HOSO	60% Ahiflower 40% HOSO	100% Ahiflower 0% HOSO
Fatty acid ^1^	%			
16:0	3.9	4.2	4.4	4.7
16:1*n*-7	0.1	0.1	0.1	0.1
18:0	2.6	2.3	2.1	1.8
18:1*n*-9	78.5	57.8	36.2	9.4
18:1*n*-7	0.8	0.8	0.7	0.6
18:2*n*-6	11.9	11.8	11.7	11.4
18:3*n*-6 (GLA)	0.0	1.5	3.0	4.9
18:3*n*-3 (ALA)	0.2	14.6	29.4	47.9
18:4*n*-3 (SDA)	0.0	5.2	10.6	17.3
20:0	0.3	0.2	0.1	0.1
20:1*n*-9	0.3	0.4	0.6	0.8
20:5*n*-3	ND	ND	ND	ND
22:0	0.8	0.6	0.4	0.1
22:1*n*-9	0.0	0.1	0.1	0.2
22:5*n*-3	ND	ND	ND	ND
22:6*n*-3	ND	ND	ND	ND
24:0	0.3	0.2	0.1	0.0
24:1*n*-9	0.0	0.0	0.1	0.1
∑ *n*-3 PUFA	0.2	19.8	40.0	65.2
∑	99.7	99.8	99.6	99.4
Fatty acid	mg per 10 mL			
SDA	0	437	893	1469
GLA	0	130	260	428
ALA	17	1302	2613	4287

^1^ Data provided by Nature’s Crops International.

**Table 2:** Baseline anthropometric and clinical measurements of enrolled participants in pure and blends of Ahiflower and high oleic sunflower oil (HOSO) groups. BMI = body mass index; HR = heart rate; LDL-C = low density lipoprotein cholesterol; HDL-C = high density lipoprotein cholesterol; SEM = standard error of the mean.

	0% Ahiflower 100% HOSO		30% Ahiflower 70% HOSO		60% Ahiflower 40% HOSO		100% Ahiflower 0% HOSO	
	$\bar{x}$	*SEM*	$\bar{x}$	*SEM*	$\bar{x}$	*SEM*	$\bar{x}$	*SEM*
*n*	24		20		20		24	
Gender								
Female	12		13		14		10	
Male	12		7		6		14	
Age (years)	27.4	*2.0*	32.2	*2.8*	36.0	*2.7*	33.6	*3.0*
Weight (kg)	72.4	*3.2*	71.0	*3.2*	76.7	*3.1*	77.0	*3.3*
BMI (kg/m^2^)	25.1	*1.0*	24.7	*1.1*	27.1	*1.3*	25.9	*1.0*
Blood pressure (mmHg)								
Systolic	111	*2*	116	*3*	117	*4*	118	*4*
Diastolic	67	*2*	68	*2*	70	*3*	74	*2*
HR (beats/min)	67	*3*	69	*3*	72	*3*	66	*3*
Triglycerides (mmol/L)	0.98	*0.11*	1.08	*0.11*	1.18	*0.14*	1.13	*0.13*
Total cholesterol (mmol/L)	4.25	*0.17*	4.39	*0.19*	4.48	*0.18*	4.50	*0.20*
LDL-C (mmol/L)	2.31	*0.15*	2.52	*0.17*	2.62	*0.16*	2.64	*0.15*
Non HDL-C (mmol/L)	2.74	*0.18*	3.01	*0.18*	3.16	*0.20*	3.16	*0.18*
HDL-C (mmol/L)	1.50	*0.08*	1.39	*0.08*	1.32	*0.08*	1.35	*0.05*
Glucose (mmol/L)	4.97	*0.07*	4.95	*0.07*	5.06	*0.07*	5.25	*0.07*

Data on intent-to-treat cohort presented. A one-way analysis of variance followed by a Tukey’s multiple comparison test was performed on age, weight and BMI data. There were no significant differences between groups.

**Table 3:** Plasma *n*-3 PUFA content (% of total fatty acids) before and after 28-day dietary supplementation with different dosages of Ahiflower and HOSO oils.

	0% Ahiflower 100% HOSO		30% Ahiflower 70% HOSO		60% Ahiflower 40% HOSO		100% Ahiflower 0% HOSO	
	Baseline	Day 28	Baseline	Day 28	Baseline	Day 28	Baseline	Day 28
**Fatty acid**	(g/100 g fatty acids)							
18:3 *n*-3	0.82 ± 0.04	0.80 ± 0.04 ^a^	0.80 ± 0.06	0.97 ± 0.08 *^,b^	0.90 ± 0.07	1.45 ± 0.08 *^,c^	0.89 ± 0.05	1.88 ± 0.14 *^,d^
18:4 *n*-3 ^1^	0.03 ± 0.02	0 ^a^	0.02 ± 0.01	0.10 ± 0.02 ^b^	0.01 ± 0.01	0.21 ± 0.03 ^b,c^	0	0.28 ± 0.05 ^c^
20:4 *n*-3 ^1^	0.08 ± 0.02	0.09 ± 0.01 ^a^	0.08 ± 0.02	0.18 ± 0.02 ^a^	0.11 ± 0.03	0.38 ± 0.07 ^b^	0.09 ± 0.02	0.43 ± 0.04 ^b^
20:5 *n*-3	0.49 ± 0.05	0.46 ± 0.04 ^a^	0.60 ± 0.05	0.97 ± 0.08 *^,b^	0.63 ± 0.06	1.32 ± 0.13 *^,b,c^	0.67 ± 0.05	1.64 ± 0.14 *^,c^
22:5 *n*-3	0.92 ± 0.08	0.87 ± 0.08 ^a^	0.91 ± 0.07	1.02 ± 0.08 ^a,b^	0.94 ± 0.09	1.12 ± 0.09 *^,b^	1.15 ± 0.10	1.45 ± 0.07 *^,c^
22:6 *n*-3	1.34 ± 0.10	1.33 ± 0.09 ^a^	1.73 ± 0.09	1.75 ± 0.09 ^a^	1.46 ± 0.07	1.44 ± 0.06 ^a^	1.54 ± 0.11	1.54 ± 0.10 ^a^
20:3 *n*-6	1.87 ± 0.08	1.80 ± 0.08	1.85 ± 0.11	1.76 ± 0.11	2.02 ± 0.15	1.98 ± 0.17	1.91 ±0.01	1.88 ± 0.07

Values are means ± SEM. Linear mixed models (LMM) were fitted to identify significant differences between treatments. Groups with different letters (a, b, c, d) are significantly different (*p* ≤ 0.05), * *p* ≤ 0.05 vs. baseline values. ^1^ A one-way analysis of variance followed by a Tukey’s multiple comparison test was performed on the change in SDA and eicosatetraenoic acid (ETA) between days 0 and 28.

**Table 4:** Mononuclear cell *n*-3 PUFA content (% of total fatty acids) before and after 28-day dietary supplementation with different dosages of Ahiflower and HOSO oils.

	0% Ahiflower 100% HOSO		30% Ahiflower 70% HOSO		60% Ahiflower 40% HOSO		100% Ahiflower 0% HOSO	
	Baseline	Day 28	Baseline	Day 28	Baseline	Day 28	Baseline	Day 28
**Fatty acid**	(g/100 g fatty acids)							
18:3 *n*-3	0.22 ± 0.02	0.23 ± 0.01 ^a^	0.23 ± 0.02	0.26 ± 0.03 ^a^	0.24 ± 0.03	0.31 ± 0.03 *^,a,b^	0.27 ± 0.02	0.40 ± 0.02 *^,b^
18:4 *n*-3 ^1^	0.01 ± 0.00	0 ^a^	0	0 ^a^	0	0.02 ± 0.01 ^a^	0	0.07 ± 0.04 ^b^
20:4 *n*-3 ^1^	0.07 ± 0.01	0.06 ± 0.01 ^a^	0.06 ± 0.01	0.10 ± 0.02 ^a^	0.08 ± 0.01	0.18 ± 0.0 ^b^	0.04 ± 0.01	0.25 ± 0.02 ^c^
20:5 *n*-3	0.25 ± 0.02	0.20 ± 0.02 *^,a^	0.29 ± 0.02	0.39 ± 0.02 *^,b^	0.29 ± 0.02	0.55 ± 0.04 *^,b,c^	0.30 ± 0.01	0.70 ± 0.05 *^,c^
22:5 *n*-3	2.36 ± 0.09	2.15 ± 0.07 *^,a^	2.35 ± 0.08	2.68 ± 0.10 *^b^	2.51 ± 0.12	3.21 ± 0.12 *^,c^	2.46 ± 0.09	3.30 ± 0.15 *^,c^
22:6 *n*-3	1.75 ± 0.09	1.64 ± 0.08 *^,a^	1.98 ± 0.11	1.90 ± 0.11 ^b^	1.90 ± 0.08	1.71 ± 0.07 *^,a^	1.82 ± 0.09	1.63 ± 0.08 *^,a^
20:3 *n*-6	2.20 ± 0.08	2.16 ± 0.09 ^a^	1.84 ± 0.12	1.94 ± 0.14 ^a^	2.22 ± 0.16	2.33 ± 0.16 ^a,b^	1.92 ± 0.08	2.14 ± 0.09 *^,b^

Values are means ± SEM. Linear mixed models (LMM) were fitted to identify significant differences between treatments. Groups with different letters (a, b, c) are significantly different (*p* ≤ 0.05), * *p* ≤ 0.05 vs. baseline values. ^1^ A one-way analysis of variance followed by a Tukey’s multiple comparison test was performed on the change in SDA and ETA between days 0 and 28.

## Supplementary Materials

The following are available online at http://www.mdpi.com/2072-6643/9/3/261/s1, Table S1: Inclusion and exclusion criteria for determining eligibility of screened subjects into the study, Table S2: Plasma *n*-3 fatty acid concentrations (µmol/L) after 28-day dietary supplementation with different dosages of Ahiflower and HOSO oils, Flow Diagram: CONSORT 2010 Flow Diagram, Checklist: CONSORT 2010 checklist.
