# Supplementary material for: Dietary Buglossoides Arvensis Oil Increases Circulating n-3 Polyunsaturated Fatty Acids in a Dose-Dependent Manner and Enhances Lipopolysaccharide-Stimulated Whole Blood Interleukin-10—A Randomized Placebo-Controlled Trial
Source: Nutrients. 2017 Mar 10;9(3):261. doi: 10.3390/nu9030261 (PMC5372924; doi:10.3390/nu9030261)
Supplement: Supplementary file 1 [file nutrients-09-00261-s001.zip › nutrients-176517-supplementary/Consort-2010-flow-diagram_Lefort_2017.docx]

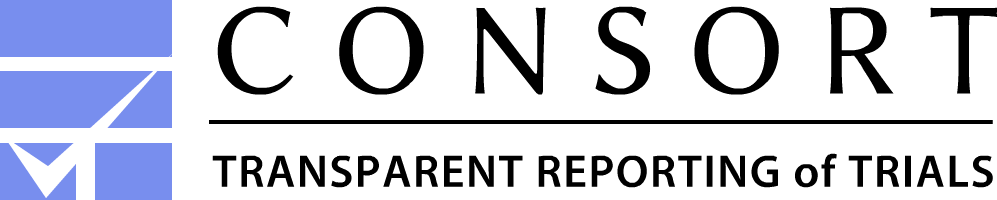


**CONSORT 2010 Flow Diagram**

## Follow-Up

Analysed (n=21)
♦ Excluded from analysis (n=3, 1 withdrawal, 2 non-compliant with dosage)

## Analysis

Analysed (n=17)
♦ Excluded from analysis (n=3, 3 non-compliant with dosage)

Lost to follow-up (n=0)

Discontinued intervention (n=0)

## Enrollment

Allocated to 100% HOSO (n=24)

♦ Received allocated intervention (n=24)

♦ Did not receive allocated intervention (n=0)

## Allocation

Allocated to 30% Ahiflower (n=20)

♦ Received allocated intervention (n=20)

♦ Did not receive allocated intervention (n=0)

Randomized (n=88)

Excluded (n=76)

♦  Not meeting inclusion criteria (n=76)

♦  Declined to participate (n=0)

♦  Other reasons (n=0)

Assessed for eligibility (n=164)

**See next page for 60% and 100% Ahiflower groups**

**CONSORT 2010 Flow Diagram**

Lost to follow-up (n=0)

Discontinued intervention (n=1, serious AE unrelated to consumption of dietary oil)

## Follow-Up

Analysed (n=15)
♦ Excluded from analysis (n=5, 2 withdrawals, 3 non-compliant with dosage)

## Analysis

Analysed (n=19)
♦ Excluded from analysis (n=5, 2 withdrawals, 3 non-compliant with dosage)

Lost to follow-up (n=0)

Discontinued intervention (n=2, 2 withdrawals)

Lost to follow-up (n=1)

Discontinued intervention (n=0)

Allocated to 60% Ahiflower (n=20)

♦ Received allocated intervention (n=19)

♦ Did not receive allocated intervention (n=1, 1 withdrawal)

## Allocation

Allocated to 100% Ahiflower (n=24)

♦ Received allocated intervention (n=24)

♦ Did not receive allocated intervention (n=0)

**(continued from previous page)**
