# Supplementary material for: Dietary Buglossoides Arvensis Oil Increases Circulating n-3 Polyunsaturated Fatty Acids in a Dose-Dependent Manner and Enhances Lipopolysaccharide-Stimulated Whole Blood Interleukin-10—A Randomized Placebo-Controlled Trial
Source: Nutrients. 2017 Mar 10;9(3):261. doi: 10.3390/nu9030261 (PMC5372924; doi:10.3390/nu9030261)
Supplement: Supplementary file 1 [file nutrients-09-00261-s001.zip › nutrients-176517-supplementary/nutrients-176517-supplementary.docx]

Supplementary Materials: Dietary Buglossoides arvensis Oil Increases Circulating n-3 PUFA in a Dose-Dependent Manner and Enhances LPS-Stimulated Whole Blood Interleukin-10—A Randomized Placebo-Controlled Trial

Natalie Lefort, Rémi LeBlanc and Marc E. Surette

**Table S1.** Inclusion and exclusion criteria for determining eligibility of screened subjects into the study.

| **Inclusion Criteria** |
| --- |
| 1. Have been using an effective form of birth control for at least 3 months prior to entry into the study and continue to do so during participation in the study. |
| 2. Be between 18 to 65 years of age, inclusive. |
| 3. Have a body mass index (BMI) 18–39.9 kg/m^2^ |
| 4. Be willing to avoid alcohol consumption for 24 h prior to every clinic visit. |
| 5. Must not smoke more than 20 cigarettes a day, and if a smoker, must not modify smoking habits during the supplementation period. |
| 6. Not have any significant medical conditions that in the opinion of the qualified physician, would preclude the subject’s participation in the study. |
| 7. Be willing to follow all study procedures, including study visits, fasting blood draws, stable body weight, normal eating habits, current activity level, and compliance with study preparation. |
| 8. Be willing to not consume fish, crustaceans and shellfish for the duration of the study. |
| **Exclusion Criteria** |
| 1. Pregnancy or lactation. Trying to conceive and unwilling to commit to the use of a medically approved form of contraception throughout the study period. |
| 2. A condition the study physician believes would interfere with the participant’s ability to provide informed consent, comply with his responsibilities during the study, which might confound the interpretation of the study results or put the person at undue risk. |
| 3. Medical conditions including an active peptic ulcer, inflammatory bowel disease, or gastrointestinal bleeding and any medical condition or prior gastrointestinal surgery that could influence absorption, metabolism or excretion of the study supplement. |
| 4. A history or presence of significant, renal, hepatic, gastrointestinal, pulmonary, biliary, neurological or endocrine disorders. |
| 5. A history or presence of cancer in the past 2 years, except for non-melanoma skin cancers (e.g. basal or squamous cell carcinoma of the skin). |
| 6. Clinically significant abnormal laboratory test results including but not limited to low density lipoprotein (LDL)-cholesterol ≥ 4.1 mM, triglyceride levels ≥3.95 mM, fasting creatinine ≥ 1.5 mg/dL, alanine aminotransferase or aspartate aminotransferase (ALT or AST) ≥ 1.5X the upper limit of normal. |
| 7. Currently being treated for angina, arrhythmia and/or congestive heart failure. |
| 8. A history of myocardial infarction or stroke. |
| 9. Uncontrolled hypertension (resting systolic blood pressure (BP) ≥ 160 mmHg or diastolic BP ≥ 100 mmHg). |
| 10. Type 1 or Type 2 diabetes. hemoglobin A1c (HbA1c) ≥6.0 |
| 11. A history of (within 12 months) or current alcohol or substance abuse (no more than 14 units per week; 1 unit = 12 oz beer, 5 oz wine, 1.5 oz distilled spirits). |
| 12. Using lipid-altering medications (statins, bile acid sequestrants, cholesterol absorption inhibitors, fibrates, and prescription formulations of niacin). |
| 13. Using anti-inflammatory medications (including asthma, allergy and pain medications) on a daily basis. Unwilling to refrain from taking anti-inflammatory medications during the three days preceding Visits 2 and 4. |
| 14. Unstable use of thyroid medication. Stable, treated hypothyroidism is OK. |
| 15. Use of a weight loss or lipid metabolism medication/supplement/program (including lipase inhibitors) within 1 month of the study period OR having gained/lost > 2 kg in the past 3 months. |
| 16. Having taken fish oil or any other omega-3 or omega-6 polyunsaturated fatty acid (PUFA) supplements/drugs within one month of Visit 1 and/or intention to take some during the study. Consumption of fatty fish (salmon, herring, mackerel, albacore tuna, and sardines) more than twice a month within one month of visit 1 and/or intention to consume some during the study period. Consumption (more than twice a month) of EPA/DHA enriched foods (e.g. DHA-enriched eggs) within one month of Visit 1 and/or intention to consume some during the study period. Patient unwilling to avoid all fish including shellfish and crustaceans during the study period. |
| 17. Use of alpha-linolenic acid-containing seeds and oils such as flax seed, perilla seed, hemp, spirulina, walnut, mustard seed or black currant seeds/oil within one month of Visit 1 and/or throughout the study. |
| 18. Used an investigational product within the previous 30 days. |
| 19. Donation of blood in the 4 weeks before the start of the study. Not willing to cease being a blood donor during the study. |

**Table S2.** Plasma n-3 fatty acid concentrations (µmol/L) after 28-day dietary supplementation with different dosages of Ahiflower and HOSO oils. HOSO = high oleic sunflower oil.

|  | **0% Ahiflower**  **100% HOSO** | | **30% Ahiflower**  **70% HOSO** | | **60% Ahiflower**  **40% HOSO** | | **100% Ahiflower**  **0% HOSO** | |
| --- | --- | --- | --- | --- | --- | --- | --- | --- |
|  | Baseline | Day 28 | Baseline | Day 28 | Baseline | Day 28 | Baseline | Day 28 |
| **Fatty acid** | (µmol/L) | | | | | | | |
| 18:3 n-3 | 79 ± 6 | 75 ± 6 ^a^ | 71 ± 7 | 77 ± 8 ^a^ | 82 ± 8 | 128 ± 9 *^,b^ | 80 ± 6 | 179 ± 19 *^,b^ |
| 18:4 n-3 ^1^ | 1 ± 0 | 0 ^a^ | 2 ± 1 | 8 ± 2 ^b^ | 2 ± 1 | 18 ± 3 ^c^ | 1 ± 0 | 28 ± 5 ^c^ |
| 20:4 n-3 ^1^ | 8 ± 2 | 8 ± 1 ^a^ | 7 ± 2 | 14 ± 2 ^b^ | 10 ± 2 | 32 ± 7 ^c^ | 7 ± 1 | 37 ± 4 ^c^ |
| 20:5 n-3 | 44 ± 5 | 39 ± 4 ^a^ | 51 ± 5 | 74 ± 9 *^, b^ | 54 ± 6 | 111 ± 14 *^, c^ | 54 ± 4 | 142 ± 17 *^,c^ |
| 22:5 n-3 | 71 ± 7 | 65 ± 6 ^a^ | 69 ± 6 | 75 ± 8 ^a^ | 72 ± 7 | 83 ± 7 ^a,b^ | 84 ± 5 | 112 ± 7 *^,b^ |
| 22:6 n-3 | 109 ± 7 | 100 ± 5 ^a^ | 135 ± 10 | 129 ± 13 ^a^ | 113 ± 6 | 108 ± 6 ^a^ | 113 ± 7 | 118 ± 7 ^a^ |

Values are means ± standard errors of the mean (SEM). Linear mixed models (LMM) were fitted to identify significant differences between treatments. Groups with different letters (a,b,c) are significantly different (*p* ≤ 0.05), * *p* ≤ 0.05 vs. baseline values. ^1^ A one-way analysis of variance followed by a Tukey’s multiple comparison test was performed on the change in SDA (18:4 n-3) and eicosatetraenoic acid (20:4 n-3) between days 0 and 28.
